# Shotgun Proteomics as a Powerful Tool for the Study of the Proteomes of Plants, Their Pathogens, and Plant–Pathogen Interactions

**DOI:** 10.3390/proteomes10010005

**Published:** 2022-01-19

**Authors:** Sadegh Balotf, Richard Wilson, Robert S. Tegg, David S. Nichols, Calum R. Wilson

**Affiliations:** 1 New Town Research Laboratories, Tasmanian Institute of Agriculture, University of Tasmania, New Town, TAS 7008, Australia; sadegh.balotf@utas.edu.au (S.B.); robert.tegg@utas.edu.au (R.S.T.); 2 Central Science Laboratory, University of Tasmania, Hobart, TAS 7001, Australia; d.nichols@utas.edu.au

**Keywords:** mass spectrometry, shotgun proteomics, plant–pathogen interaction, post-translational modification

## Abstract

The interaction between plants and pathogenic microorganisms is a multifaceted process mediated by both plant- and pathogen-derived molecules, including proteins, metabolites, and lipids. Large-scale proteome analysis can quantify the dynamics of proteins, biological pathways, and posttranslational modifications (PTMs) involved in the plant–pathogen interaction. Mass spectrometry (MS)-based proteomics has become the preferred method for characterizing proteins at the proteome and sub-proteome (e.g., the phosphoproteome) levels. MS-based proteomics can reveal changes in the quantitative state of a proteome and provide a foundation for understanding the mechanisms involved in plant–pathogen interactions. This review is intended as a primer for biologists that may be unfamiliar with the diverse range of methodology for MS-based shotgun proteomics, with a focus on techniques that have been used to investigate plant–pathogen interactions. We provide a summary of the essential steps required for shotgun proteomic studies of plants, pathogens and plant–pathogen interactions, including methods for protein digestion, identification, separation, and quantification. Finally, we discuss how protein PTMs may directly participate in the interaction between a pathogen and its host plant.

## 1. Introduction

As a multifaceted process, plant–pathogen interactions have been extensively researched from both the pathogen and plant viewpoints. Plants are surrounded by many microorganisms, some of which can cause diseases and lead to negative impacts on yield, quality, and value [1]. Pathogen-derived molecules such as nucleic acids, secondary metabolites, and proteins are major factors in pathogenicity which allow the successful invasion and colonization of host tissues. Plants also produce molecules important in recognition of the pathogens and pathogen-derived molecules that can elicit defense responses. These responses need to be quick and specific to minimize the damage caused by pathogenic microorganisms [2]. An understanding of how pathogens and plants recognize each other and how they communicate pre- and post-infection is crucial in this field of investigation. The increase in genomic and transcriptome studies has advanced the understanding of the pathogenicity strategies employed by pathogens and the immune responses in plants [2,3,4,5]. However, these approaches have limitations and cannot monitor post-transcriptional processes [6]. Therefore, investigation at post-transcriptome levels (i.e., proteome and metabolome levels) is required for a better understanding of the pathogen–host interaction.

Proteomics is defined as the large-scale study of different proteins expressed by an organism [7] and has become a driver in discovering host-pathogen communication [8]. Study at the proteome level allows the concurrent study of the total proteome, its qualitative presence and quantitative abundance, variation within a population, and localization. The role of mass spectrometry (MS) in different aspects of plant biology, such as plant defense and signal transduction, has been reviewed in detail [9,10]. Technological advances in MS-based proteomics have significantly accelerated the characterization of pathogen–host protein interactions [11] and while gel-based methods for intact protein separation and identification remain in use for specialized applications [12], shotgun analysis is well established as the dominant proteomics platform [13]. The success of these approaches depends on various factors, including the methods used for isolation, digestion, separation, identification, and quantification of proteins [14]. In addition to improvements in MS sensitivity that continue to extend proteome depth, modern tools for bioinformatic analysis [15] and the availability of annotated genomes for many non-model organisms [16,17,18,19,20] has increased the potential to expand our understanding of plant–pathogen interaction. The purpose of this review is to summarize the research techniques of proteomics and posttranslational modification (PTM) analysis that can be used to uncover regulatory principles underlying plant host-pathogen interaction. We discuss methodological aspects, with emphasis on sample preparation, MS strategies, and PTMs, with a focus on the approaches that have already been successfully introduced in plant–pathogen studies.

## 2. Sample Preparation Prior to LC-MS/MS

An efficient sample preparation method for obtaining high-quality peptides for proteomic analysis of pathogens and plants represents a greater challenge than most other cell/tissue types [21]. Plant tissues with robust cell walls can be difficult to fully disrupt and contain relatively high levels of secondary metabolites that can interfere with proteome analysis [22]. In pathogens, sample preparation for proteomics can also be challenging. For example, obligate biotrophic pathogens are not culturable on the artificial media, and therefore it can be difficult to obtain pure samples of these pathogens [23]. In addition, many plant pathogens such as soilborne pathogens, produce long-lived resting spores, which are highly resistant to adverse environmental conditions [24]. Harsh conditions, including mechanical force, must be applied for the disruption of the thickened cell walls in these structures [25].

### 2.1. Protein Extraction

Protein extraction is one of the most critical steps in proteome analysis studies [26]. The ideal extraction method should first and foremost be robust in terms of reproducibility. Extraction conditions should minimize protein degradation and unwanted modifications, and solubilize the maximum number of proteins [27]. Plant tissues contain large amounts of compounds, including phenolics, organic acids, pigments, and polysaccharides that interfere with further steps of protein analysis [28]. There are several different methods to disrupt microorganisms and plant tissues and reduce protein degradation. Cell disruption for protein extraction can use chemical and/or physical approaches [29]. Snap freezing in liquid nitrogen, bead beating, the addition of organic solvents, thermolysis, and sonication are commonly used in plant and pathogen proteomics studies. Selecting the best cell/tissue disruption method depends on several factors and has been extensively reviewed before [27,30,31,32,33]. A perennial challenge for proteomic analysis of plant tissues is the interference of high abundance proteins such as the subunits of ribulose-1,5-bisphosphate carboxylase oxygenase (Rubisco), which can account for up to 50% of total protein in mature leaves of C3 plants [34]. Depletion of Rubisco is therefore potentially one method to extend proteome coverage by “unmasking” lower abundance proteins. Several methods have been developed for the depletion of highly abundant proteins [35,36,37], of which a few examples are presented here. Widjaja et al. [38] used a combination of Rubisco depletion and sub-proteome enrichment for the identification of low abundance proteins during Arabidopsis defense response. This approach enabled them to identify several low abundance proteins that differentially regulated post infection. Zhang et al. [39] developed a polyethyleneimine assisted Rubisco cleanup (PARC) method to improve proteomics coverage in rice plants. The results showed that PARC effectively removed Rubisco and improved protein identification. In another study of the plant proteome, a fractionation method using 10 mM of Ca^2+^ and 10 mM of phytate was used to precipitate Rubisco from soybean leaf protein extract [40]. This technique successfully removed 85% Rubisco enzyme from soybean leaf extract and enable identification of several new low abundance proteins. 

### 2.2. Sample Cleanup 

Plant and pathogen proteomics has currently moved from gel-based methods to gel-free shotgun (bottom-up) approaches [41,42,43]. Several new strategies for proteome sample preparation involving the removal of substances such as detergents, salts, and chaotropic agents which interfere with the protein digestion and MS analysis were introduced in recent years [44]. Traditional approaches for the removal of surfactants and other contaminants have been reviewed before [45,46]. More recent innovations include filter-aided sample preparation (FASP) [47], protein suspension trapping (S-Trap) [48], and single-pot solid-phase-enhanced sample preparation (SP3) [49]. In the FASP method, the sample lysate is applied to an ultrafiltration unit for washing out the low-mass contaminants and digesting the proteins on the membrane. Although many useful modifications of the classical FASP protocol have been published [47,50,51,52], there are a few disadvantages with the FASP method, which can limit the application of this method. The FASP methods are expensive and rather time-consuming. Moreover, the efficiency of the FASP filter for SDS removal has been doubted by the detection of traces of remaining SDS after FASP [53]. In the S-Trap (suspension trapping) method, the proteins are digested in a filter after removing contaminants in a short wash step. In contrast to the FASP method, which employs a molecular weight cut-off membrane, the S-Trap filter consists of a three-dimensional porous material. FASP requires hours of processing (approximately 3 h), while due to the large pore size, the total processing time is reduced to less than 15 min in the S-Trap [54]. In the protein analysis of bacterial whole-cell lysate, both S-trap and FASP methods yielded similar results regarding peptide and protein identifications [54]. The SP3 protocol consists of nonselective protein binding, where proteins are captured on the surface of magnetic beads. The beads are compatible with various organic solvents and detergents, including urea, SDS, and acetonitrile (ACN). The adaptability of the SP3 protocol to a 96-well platform provides a fast and efficient technique easily applicable for large-scale protein interactome analysis [55,56]. Ludwig et al. [57] also showed that S-Traps outperformed FASP and in-solution digest methods for colorectal cancer cell lysate regardless of lysis conditions. However, the potential for losses during wash steps if protein material does not completely aggregate onto magnetic beads remains the main limitation to the SP3 protocol. The solvent precipitation SP3 (SP4) method can be an efficient and effective alternative to SP3 [58], in which the magnetic beads are omitted, and brief centrifugation with or without an inert glass bead capture the aggregated protein. SP4 recovered equivalent or greater protein yields and improved reproducibility compered to SP3. 

The selection of sample preparation method is highly dependent on tissue type, and no sample preparation method has been found to be applicable for all sample types. For example, Mikulášek et al. [59] showed that the SP3 workflow was the best sample preparation method (in comparison to FASP and S-Trap) for protein analysis of Arabidopsis leaves in terms of number of identifications, proteome coverage, number of missed cleavages, reduction of handling time, repeatability, and cost per assay. Similarly, Stoychev [60] found that the SP3 workflow resulted in over 30% increase in identified post-translational modifications of peptides and an approximately two-fold increase in peptide recovery compared to FASP. In contrast, the SP3 method was not very successful for the proteomic analysis of barley anthers [61]. In the protein analysis of resting spores in the obligate biotrophic plant pathogen *Spongospora subterranea*, the S-Trap method delivered a higher number of protein identifications with an improved reproducibility compared to the SP3 method [24,62]. 

## 3. MS Strategies

There are many options for MS-based proteomics, and decisions on what approaches to use are influenced by the equipment and expertise available as well as the specific research questions [63]. An untargeted proteome analysis that provides global-scale proteome changes would likely be chosen by the researcher aiming to quantify as many proteins as possible [64]. For the accurate quantification of a specific protein or small group of proteins, a targeted proteomics approach is preferred [65]. Deciding on the data acquisition and quantification methods is highly dependent on the experimental design and sample preparation [66]. 

Mass spectrometry using data-dependent acquisition (DDA) and, increasingly, data-independent acquisition (DIA) approaches have dominated the methodology for untargeted proteomics [67]. In DDA mode, the N most intense peptide precursors in a survey MS1 scan (10–25 most abundant peptides) at each point of the chromatographic gradient are identified and fragmented to acquire MS2 spectra. Each MS2 spectrum in DDA is effectively a single analyte and is matched to a protein database to identify specific peptides [68]. The well-established instrument operation, the option of label-dependent quantitation, data analysis, and processing pipelines are all benefits of DDA workflows [69,70]. However, due to the semi-stochastic sampling of lower abundance peptides, the inter-sample reproducibility in DDA is relatively low. The resulting “missing value” problem potentially limits the statistical analysis of all identified proteins across an experiment [64]. DIA methods represent an appealing alternative for DDA as all theoretical peptides in a sample are fragmented sequentially across mass windows of predefined m/z intervals. This provides quantitative data across the chromatographic peak at the MS2 level, which can be used in addition to MS1-level data for more precise peptide quantitation [71,72]. While the resulting MS2 spectra are highly multiplexed, an ever-expanding array of software solutions exist for MS2 spectrum deconvolution and peptide identification, using both spectrum-centric and peptide-centric approaches [73,74,75,76]. In addition to improvements in sensitivity and reproducibility, a further benefit of the DIA workflow is the ability to reanalyze previous DIA results as spectral libraries and algorithms are developed [77]. Parallel accumulation–serial fragmentation (PASEF) is another acquisition method that enhances sequencing speed and enables hundreds of MS/MS events per second at full sensitivity. In this method, synchronized scans in a trapped ion mobility device allow a 10-fold gain in sequencing speed without decreasing sensitivity [78]. A detailed description of the construction and operation of the PASEF has been published elsewhere [79,80,81]. This approach can improve protein identification in a complex interaction between plant and pathogen. Jin et al. [82] used a PASEF-MS/MS workflow to identify proteins associated with *Fusarium crown* rot resistance in wheat. A total of 9234 proteins were identified, including proteins associated with defense, photosynthesis, and cell wall formation. 

**Figure 1:**
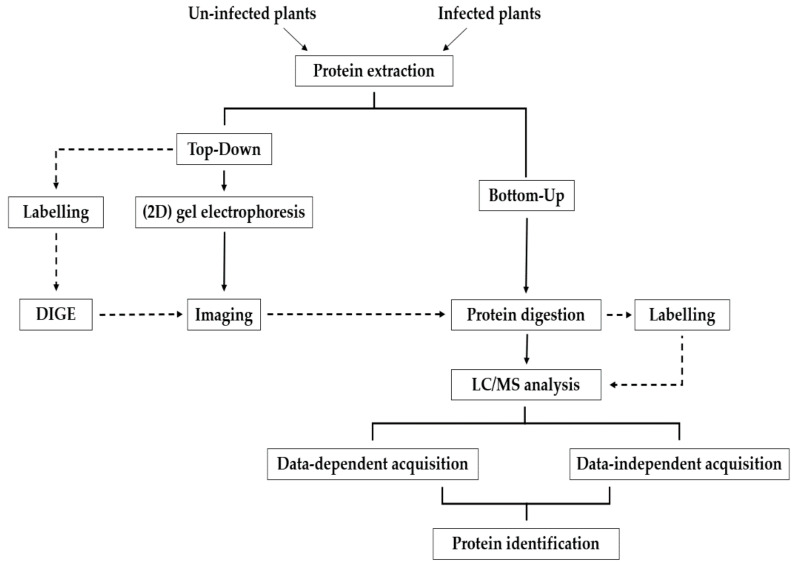
Overview of different experimental workflows for shotgun proteomics.

Proteomic approaches can also be classified as label-based or label-free, which both have their own sets of strengths and limitations [83]. For intact protein analysis, the two-dimensional difference gel electrophoresis (2D-DIGE) method [84] uses protein labeling with cyanine fluorescent dyes for relative protein quantitation between two or more multiplexed samples. 2D-DIGE has been widely used in the study of plant proteomes [85,86,87] but mostly prior to the more widespread adoption of gel-free proteomics. In another early example of label-based analysis, this time analogous to the SILAC approach, Bindschedler et al. [88] developed a cost-effective method called hydroponic isotope labeling of entire plants (HILEP) for quantitative plant proteomics. In HILEP, the whole and mature plants are labeled with a stable isotope such as ^15^N. Zhang et al. [89] later used HILEP combined with phosphopeptide enrichment to study the phosphorylation events in auxin signaling in lateral root induction of Arabidopsis. The isobaric tag for relative and absolute quantitation (iTRAQ) is another label-based method that is used in plant proteomics [90], pathogen proteomics [91], and plant–pathogen interaction analysis [92]. One of the advantages of iTRAQ compared to HILEP is that iTRAQ allows for 4 or 8 comparisons, while HILEP is suitable for pair-wise comparisons [87]. In label-based quantitation, most often used in conjunction with DDA-MS, samples are differentially labeled with alternative differential mass tags, which allows the detection of peptides based on the change in the mass [64,93]. Sample multiplexing reduces variability, which can substantially minimize instrument time if “single shot” analysis is used. Alternatively, peptide labeling can be combined with off-line fractionation using an orthogonal separation approach (e.g., strong cation exchange) as a method to significantly extend proteome depth. The proteolytic, metabolic, and chemical labeling strategies are the most widely used labeling methods [94] and can be used for both absolute and relative quantification of proteins [95]. In contrast to label-based methods, label-free quantification of peptides is typically a more straightforward workflow that also does not require expensive labeling reagents. An overview of the experimental workflows for methods that can be used in shotgun proteomics is presented in Figure 1. 

Several recent studies have employed DIA approaches for the analysis of the interaction between pathogens and their host plants. A DIA-MS workflow was used to profile the proteome of *S. subterranea* in resistant and susceptible potato cultivars [96]. The finding of this study illuminated the regulatory principles underlying *Spongospora*–potato interaction. The interaction between barley and *Pyrenophora teres* was also studied using DIA-MS [97]. Over 1000 proteins were quantified in which the increase in abundance of several classes of pathogenesis-related (PR) proteins was confirmed. A similar method demonstrated an increase in jasmonic acid biosynthesis and a decrease in photosynthesis-associated proteins in rice plants in response to pathogen infection [98]. More than 2000 proteins from tomato leaves infected by *Pseudomonas syringae* were identified using DIA-MS [99]. The significantly changed proteins belonged to immune response, redox processes, energy generation, and carbon fixation in the chloroplast. Fan et al. [100] employed a DIA-MS method to study the interaction between tomato and the hemibiotrophic oomycete pathogen *Phytophthora infestans*. Among the changed proteins, several were involved in plant defense responses, metabolic pathways, and signaling. The effect of *Funneliformis mosseae* in soybean roots was investigated using a transcriptomic and proteomic (DDA-MS) analysis. A total of 9488 proteins were identified, and the key pathways and differentially abundance proteins were involved in plant–pathogen interaction, phenylalanine metabolism, hormone signal transduction, and metabolic pathways [101]. Using a combination of a shotgun DDA-MS and a targeted DIA-MS, several peptides of potential markers for resistance to *Peyronellaea pinodes*, causing Ascochyta blight, were identified. This study revealed the importance of plant cell walls to hinder the growth of the pathogen within cells and redox response for the detoxification of fungal toxins [102]. Kerr et al. [103] used DIA-MS to analyze barley seed proteome during fungal infection. This study showed that oxalate oxidase was the only protein consistently increased in abundance in the infected plants. 

## 4. Post-Translational Modifications

All living organisms need to respond to environmental changes quickly and efficiently using a strict regulatory system through molecular interactions of hundreds to thousands of biomolecules [104]. Due to multiple levels of regulation such as PTM and alternative splicing, a single gene can produce several different proteins, increasing proteome diversity [105]. PTMs act as molecular switches that can lead to dramatic changes in the regulation of molecular functions without changes in the transcriptome and proteome levels [106]. Protein phosphorylation is one of the most frequently studied PTMs and represents over 53% of all the PTMs based on the published experimental data [107]. Phosphorylation/dephosphorylation of proteins is a fast response that can switch on or off the cell processes or biological pathways. In eukaryotes, phosphorylation occurs on serine, tyrosine, and threonine [108]. MS-based proteomics has become the primary tool used to study protein phosphorylation. The simultaneous identification/quantitation of phosphopeptides and proteins has expanded our understanding of complicated biological systems and their regulation [109,110,111,112,113].

**Figure 2:**
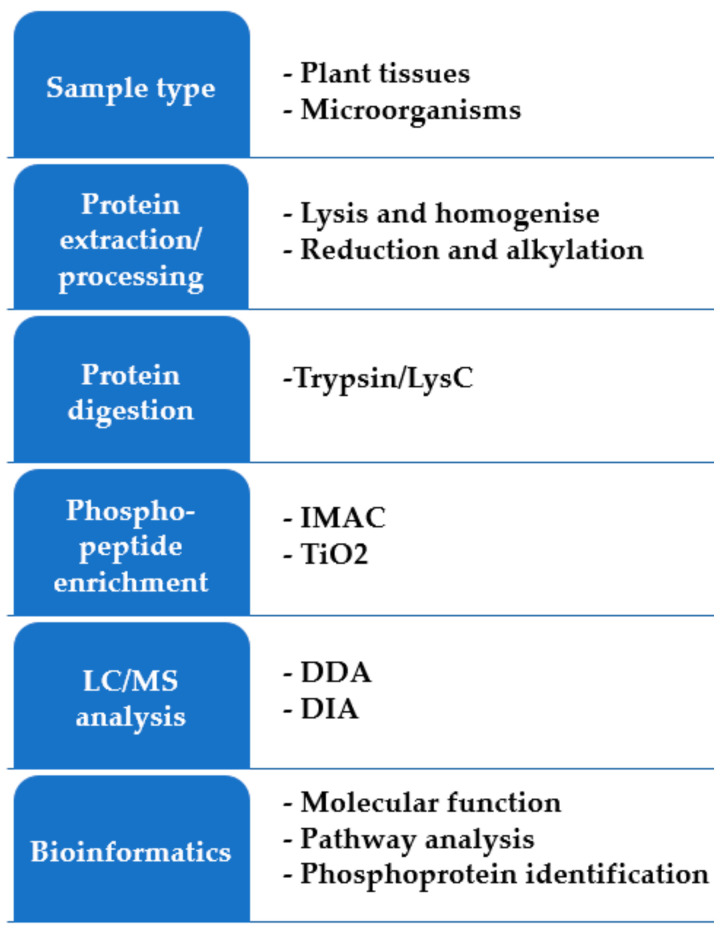
An overview of the possible experimental workflows for shotgun phosphoproteomics.

Similar to proteomics, a phosphopeptide analysis workflow starts with protein extraction and digestion (Figure 2). Phosphopeptide analysis needs one- or multi-stage enrichment strategies to achieve comprehensive coverage of phosphorylation events [114,115], and therefore requires more starting material, in the range of an order of magnitude compared with total peptide analysis. Enrichment methods include immobilized metal affinity chromatography (IMAC), metal oxide affinity chromatography (MOAC), titanium dioxide (TiO_2_) phosphopeptide enrichment, electrostatic repulsion hydrophilic interaction chromatography (ERLIC), and phosphopeptide precipitation [116,117,118,119]. Large-scale profiling of plant phosphoproteomes after infection by pathogens has revealed the dynamic phosphorylation events that regulate plant resistance or susceptibility to pathogens [110,120]. Quantitative phosphoproteomics analysis of Arabidopsis revealed the regulatory mechanisms of pathogen-associated molecular pattern immunity. The results of this study showed that some of the identified phosphosites are required for the production of reactive oxygen species during immunity against a virulent necrotrophic fungus [121]. Phosphoproteomics profiling of cotton roots in response to the soilborne plant pathogenic fungus *Verticillium dahliae* infection identified 92 and 38 specific phosphoproteins in the resistant and susceptible lines, respectively [122]. 

In addition to phosphorylation, large-scale profiling of other PTMs such as acetylation and ubiquitination during plant–pathogen interactions is becoming more common. In rice [123,124] and Arabidopsis [125,126,127], hundreds of ubiquitin-modified proteins were identified during the plant immune responses. The acetylome profiling of maize in response to *Cochliobolus carbonum* infection confirmed the hyperacetylation of several proteins, including chromatin remodeling enzymes and transcription factors [128]. Although recent studies have identified many acetylated proteins in diverse pathogens, the impact of these PTMs on the pathogenicity of plant pathogens is yet to be understood [129,130,131,132]. 

## 5. Bioinformatics

Molecular research routinely involves the application of computational methods to convert raw experimental data into condensed results for biological interpretation. In recent years, several software packages and online platforms have been developed to facilitate the analysis, interpretation, and visualization of proteomics data. The MaxQuant/Perseus platform is a user-friendly, interactive workflow environment and can provide complete documentation of computational methods, and has become one of the most popular software suites for biological interpretation of protein quantification, interaction, and PTM data [133]. Relative label-free quantification of shotgun proteomics data is one of the most used applications in biological science, including plant science, microbiology, and plant pathology. LFQ-Analyst, which is easy-to-use, has recently been created to perform differential expression analysis with “one-click” and to visualize proteomics data sets preprocessed with MaxQuant software [134]. Galaxy [135] and MetaboAnalyst [136] also provide various workflow management systems to analyze MS-based proteomics data. The PeptideWitch software package is a python-based web module for the label-free shotgun proteomics data visualization [137]. This software produces many statistical and graphical outputs including heatmaps, volcano plots, Venn diagrams, and *p*-value histograms. In addition to the technical advance in proteomics data analysis software, specific bioinformatics tools have been developed to study the relationships between plants and pathogens. For example, PhytoPath, which is a database on plant–pathogen interactions, provides genome-scale data from pathogens with information about plant infection phenotypes. To date, this database includes the genome information of 99 plants, 107 pathogens, and 350 interactions. The access to the complete assembly of genome and gene models of phytopathogens in PhytoPath gained using the Ensembl Genomes browser [138]. NIASGBdb, which links the genetic resources of plants and pathogens to plant disease information, is another example of plant–pathogen interactions databases [139].

Data sharing in MS-based proteomics is becoming a standard for proteomics researchers. However, any proteomics data set is only partially understood according to the availability of the currently available analytical tools (such as the algorithms for peak detection and quantitation and the proteome database that was searched). Therefore, there is an excellent opportunity for re-analyzing and reusing public data, particularly for benchmarking studies and evaluation of new bioinformatics software. While the majority of publicly available data sets correspond to human and other main model organisms, there has been a rapid expansion in proteomic datasets for non-model organisms in recent years [140]. The first proteomics resources were set up more than 15 years ago. The GPMDB [141], PeptideAtlas [142], and the PRoteomics IDEntifications (PRIDE Archive) [143] databases are a few examples of MS-based proteomics resources. PRIDE is the world’s largest MS-based proteomics data repository, with an average of around 500 datasets deposited per month during 2021 [144]. PRIDE stores datasets coming from all experimental proteomics approaches, including DDA and DIA proteomics. The PhosPhAt 4.0 is one of the most significant phosphorylation databases for Arabidopsis phosphorylation studies and contains phosphorylation sites identified in Arabidopsis by MS-based proteomics [145]. In addition, the PhosPhAt 4.0 database includes phosphorylation site prediction and kinase–target relationship retrieval, which provides researchers with more functionality for plant phosphoproteomics analysis. The availability of large MS datasets and bioinformatics tools has enabled the comparison of different proteomics studies. For example, Pinski et al. [146] used several previously published MS datasets to compare the bioinformatics predictions of the sub-cellular localization of cell wall proteins. Collectively, these public databases include valuable resources for research on humans, model organisms, non-model plants, and microorganisms. However, there is still a need for more comprehensive plant–pathogen genome and proteome databases [147].

## 6. Conclusions and Future Perspective

In summary, recent advances in MS-based proteomics and bioinformatics tools now enable the robust profiling of plant and pathogenic microbe samples to an unprecedented depth. Global proteome profiling using shotgun approaches during infection can identify specific proteins, molecular functions, and PTMs involved in plant disease resistance and susceptibility and pathogenicity processes. Therefore, proteomics will remain one of the fastest-growing areas in plant and pathogen research. Increasingly, studies that use “omics” technologies in combination (multi-omics approaches) enable cross-validation of data sets and the ability to filter out the most significant biological changes. Integration of shotgun proteomics with other omics approaches will further expand our understanding of biological mechanisms involved in host–pathogen interactions. 

Proteomics analysis of mixtures containing different cell populations only provides a quantitative analysis of proteins that reflect the average variation in the whole cell population. Thus, the molecular changes in distinct subpopulations of rare cells will be missed in bulk sampling methods [148]. Techniques that address single cells’ molecular identity can help better understand uniqueness within the complexity of plant–pathogen interaction. The proteome of single cells can provide unique information about the processes taking place in the interaction between plants and pathogens, revealing signaling events that are taking place in a specific types of plant (or pathogen) cells. While single cell proteomics has a huge potential in developing a better understanding of the intricate connections between the host plant and its pathogen, it has yet to be applied to the analysis of such a complex interaction. 

In addition, cross-linking mass spectrometry (XL-MS) has recently emerged to study protein interactomics on the system-wide level [149]. XL-MS is a unique technology capable of capturing the dynamic biological assemblies in their native environment and uncovering their physical interaction contacts [150,151]. Liu et al. [152] developed an in planta chemical cross-linking-based quantitative interactomics (IPQCX–MS) workflow in Arabidopsis to study protein–protein interactions. They identified 354 unique cross-linked peptides and showed that this workflow can identify hundreds of peptides cross-linked in vivo. Considering the exciting new developments in computational approaches, XL-MS can be expected to become one of the most versatile methods in the study of plant–pathogen interactions within the next ten years. Together, we believe that proteomic studies using the techniques presented in this review can guide breeding efforts to identify resistant cultivars and help with the development of antimicrobial agents, leading to improved global food security.
